# The *Mesorhizobium huakuii* transcriptional regulator AbiEi plays a critical role in nodulation and is important for bacterial stress response

**DOI:** 10.1186/s12866-021-02304-0

**Published:** 2021-09-12

**Authors:** Xiaohong Chen, Aiqi Hu, Qian Zou, Sha Luo, Hetao Wu, Chunlan Yan, Tao Liu, Donglan He, Xiaohua Li, Guojun Cheng

**Affiliations:** Hubei Provincial Engineering and Technology Research Center for Resources and Utilization of Microbiology, College of Life Sciences, South-Central University for Nationalities, Wuhan, 430074 Hubei China

**Keywords:** Type IV toxin–antitoxin system, *Mesorhizobium huakuii*, Transcriptional regulator AbiEi, Symbiosis nitrogen fixation, RNA-Seq analysis of nodule bacteroids

## Abstract

**Background:**

Bacterial abortive infection (Abi) systems are type IV toxin–antitoxin (TA) system, which could elicit programmed cell death and constitute a native survival strategy of pathogenic bacteria under various stress conditions. However, no rhizobial AbiE family TA system has been reported so far. Here, a *M. huakuii* AbiE TA system was identified and characterized.

**Results:**

A mutation in *M. huakuii abiEi* gene, encoding an adjacent GntR-type transcriptional regulator, was generated by homologous recombination. The *abiEi* mutant strain grew less well in rich TY medium, and displayed increased antioxidative capacity and enhanced gentamicin resistance, indicating the *abiEi* operon was negatively regulated by the antitoxin AbiEi in response to the oxidative stress and a particular antibiotic. The mRNA expression of *abiEi* gene was significantly up-regulated during *Astragalus sinicus* nodule development. The *abiEi* mutant was severely impaired in its competitive ability in rhizosphere colonization, and was defective in nodulation with 97% reduction in nitrogen-fixing capacity. The mutant infected nodule cells contained vacuolation and a small number of abnormal bacteroids with senescence character. RNA-seq experiment revealed it had 5 up-regulated and 111 down-regulated genes relative to wild type. Of these down-regulated genes, 21 are related to symbiosis nitrogen fixation and nitrogen mechanism, 16 are involved in the electron transport chain and antioxidant responses, and 12 belong to type VI secretion system (T6SS).

**Conclusions:**

*M. huakuii* AbiEi behaves as a key transcriptional regulator mediating root nodule symbiosis.

**Supplementary Information:**

The online version contains supplementary material available at 10.1186/s12866-021-02304-0.

## Background

Toxin-antitoxin (TA) systems are ubiquitous constituents found on plasmids or chromosomes of countless bacteria, archaea, and possibly also unicellular fungi [1]. Bacterial TA systems are composed of bicistronic operons encoding a stable toxin that can harm the host cell and its cognate labile antitoxin, which protects the host from the toxin’s deleterious effect [2]. The product of the toxin gene is a protein, while the product of the antitoxin is either a protein or a non-coding RNA [3]. TA systems have been shown to play various physiological roles in the formation of dormancy and persister cells, survival during infection, adaptation to hostile environments, programmed cell death and biofilm formation [4–7]. Based on the biochemical nature and mode of action of the antitoxin gene product, bacterial TA systems have been divided into six types: small regulatory RNAs in types I and III, and antitoxins of proteinaceous nature in type II, IV, V and VI TA systems [8, 9].

The proteinaceous antitoxin of type IV system neutralizes its cognate toxin by forming toxin–antitoxin complexes instead of a direct protein-protein interaction [10]. Several common families of type IV system have been identified on the chromosomes of bacteria and archaea: CbtA/CbeA, YkfI/YafW, YpjF/YfjZ, and AbiEii/AbiEi. The pair CbtA/CbeA was the first type IV system found in *E. coli*. The toxin CbtA alters cell shape by inhibiting the polymerization of cytoskeletal proteins FtsZ and MreB through direct interaction, without showing direct interaction with its cognate antitoxin [3]. The first AbiE system was reported in *Lactococcus lactis* where it acts at the post-transcriptional level in the lytic cycle to abort phage development [11]. AbiE was found to be a type IV TA system as there is no apparently direct interaction between the antitoxin and toxin, and the antitoxin antagonizes toxin activity by stabilizing its targets [12]. The AbiEii toxin is a putative nucleotidyltransferase containing a C-terminal domain involved in toxin neutralization. The AbiEi antitoxin, a transcriptional regulator, contains an N-terminal domain required for repression of *abiE* transcription, and a bi-functional C-terminal domain required for transcriptional repression and sufficient for toxin neutralization [13]. Previous work on the *Streptococcus agalactiae* AbiE system revealed that AbiEi negatively autoregulates the *abiE* promoter and is sufficient for antitoxicity, yet it is unknown whether all systems provide dual resistance functions [3, 13].

Bacteria of the family Rhizobiaceae are able to induce symbiotic nodules on the roots of leguminous plants where bacteroids convert atmospheric nitrogen to ammonia [14]. This highly complex process requires a specific signal exchanges between the partners. Compatible rhizobia sense the flavonoids released from their host roots through the transcriptional activator NodD, which trigger nodule organogenesis [15]. Earlier reports described the presence of type II TA modules in *Sinorhizobium meliloti* and *Bradyrhizobium japonicum*. The *bat*/*bto* module is classified as a type II TA system belonging to the vapBC-family, and deletion of the *B. japonicum bat/bto* operon resulted in alterations of several metabolic pathways and defective symbiotic performance due to the changes in lipopolysaccharide (LPS) [16]. Milunovic et al. reported that loss of 4 *S. meliloti* type II TA systems results in growth inhibition, but does affect the *S. meliloti*-alfalfa symbiosis [17]. However, the *S. meliloti ntrR* gene, a member of the type II TA system, was controlled negatively by its own product and positively by the symbiotic regulator *syrM*. Mutation in the *ntrR* gene induced nodules with enhanced nitrogen fixation capacity [18].

Rhizobial genomes frequently contain type IV AbiE toxin-antitoxin operator, but unlike with the type II TA system, knowledge of regulation of the type IV AbiE system in *Rhizobium* species is still poorly documented. Here, we identified a type IV TA antitoxin gene *abiEi* in *M. huakuii* 7653R, and the roles of *M. huakuii abiEi* in free-living bacteria and during N_2_-fixing symbiosis with *A. sinicus* were investigated by analyzing the phenotypes of *abiEi* mutant strain. A transcriptome analysis was also carried out to discover clues that might explain the differences in the nodules induced by the *abiEi* mutant and the wild type strain. To our knowledge, this work represents the first transcriptome analysis of TA gene in symbiotic root nodules reported to date.

## Results

### Determination of AbiE system activity

**Fig. 1:**
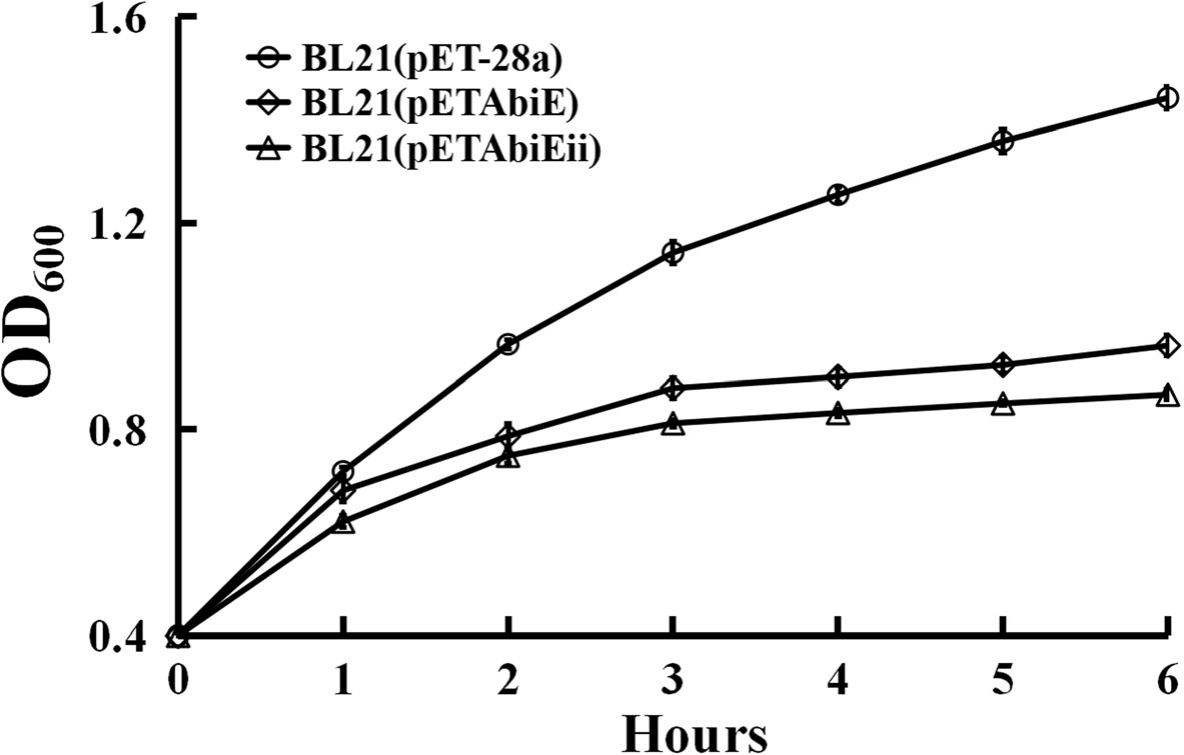
The growth curve of the *E.coli* BL21 strains after IPTG induction. *E. coli* strain BL21(pET-28a), the recombinant strains BL21 (pETAbiE) and BL21 (pETAbiEii) were cultured in LB at OD_600_ of 0.4, then induced with 1 mM IPTG. Data are from three biological samples plus and minus the standard error of the mean (± SEM)

In order to elucidate the function of the TA system, the AbiE gene cassette and the toxin gene *abiEii* was overexpressed in *E. coli*, and the effect of expressed protein product was ruled out by comparing the growth of *E. coli* BL21(DE3) cells in the presence versus the absence of the plasmid (Fig. 1). Under IPTG induction, both *E. coli* strains harboring either recombination vector pETAbiE or pETAbiEii grew more slowly than control cells with the empty vector pET-28a, which indicated that induced expression of AbiE system or AbiEii toxin protein has a negative effect on the growth of host cells. However, *E. coli* strains harboring both toxin and antitoxin genes (pETAbiE) grew slightly, but significantly faster than that harboring toxin gene *abiEii* (pETAbiEii), suggesting antitoxin AbiEi is a transcriptional regulator that can repress *abiEii* expression to influence toxin.

### Construction of *M. huakuii abiEi* mutant

**Fig. 2:**
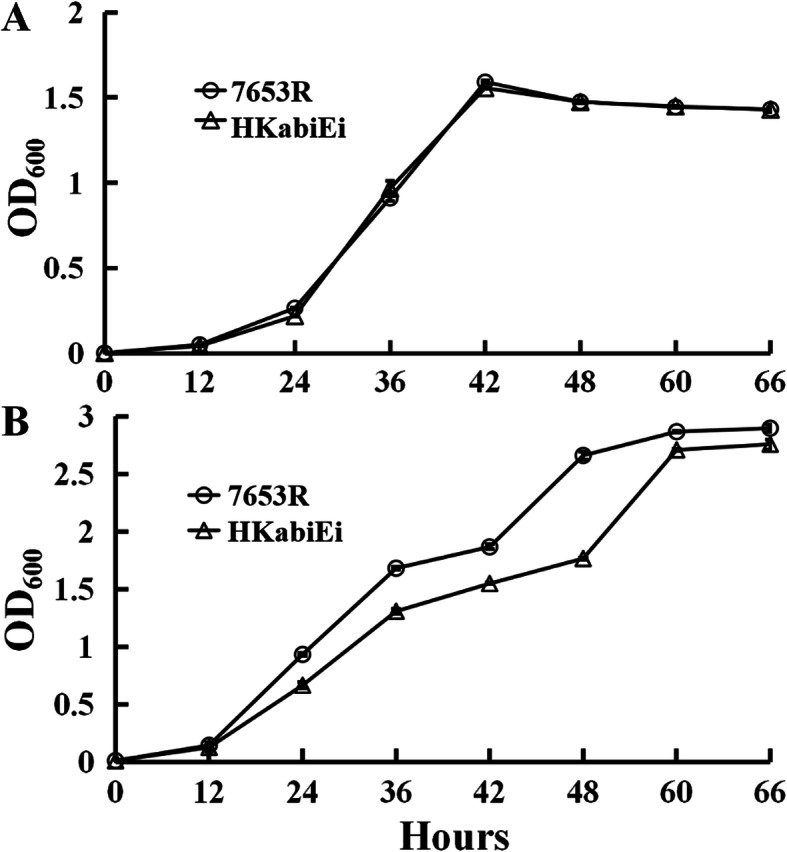
Growth of *M. huakuii* in AMS or TY media. Wild-type 7653R and mutant HKabiEi were grown in AMS supplemented with Glc/NH4^+^ (**A**) and TY (**B**). Data are from three biological samples plus and minus the standard error of the mean (± SEM)

The gene *MCHK_RS33180* in *M. huakuii* encodes a putative type IV TA system transcriptional regulator AbiEi. The *abiEi* gene is predicted to encode a 221-amino acid polypeptide with an expected molecular mass of 24.94 kDa and a pI value of 9.95. To confirm the function of the *abiEi* gene in growth, environment stress and symbiotic nitrogen fixation, a mutant HKabiEi strain of this gene was constructed by single crossover homologous recombination. qRT-PCR was firstly employed to examine the relative mRNA levels of antitoxin and toxin in free-living cells from *M. huakuii*. The expression of antitoxin gene *abiEi* was almost not detected in the mutant HKabiEi, while the expression of toxin gene *abiEii* was 0.86 ± 0.11, and there was no significant difference in the mutant HKabiEi compared to the wild-type strain 7653R. The results confirmed that *abiEi* was disrupted by the insertion of vector pK19mob in HKabiEi, and also indicated that antitoxin AbiEi didnot directly regulate toxin AbiEi expression, and may antagonize toxin activity by stabilizing its targets. The mutant showed no significant difference in AMS minimal medium with NH_4_Cl as nitrogen source and glucose as carbon source, but exhibited defective growth in rich TY medium (Fig. 2).

### Role of the *abiEi* gene in the regulation of antibiotic resistance

**Fig. 3:**
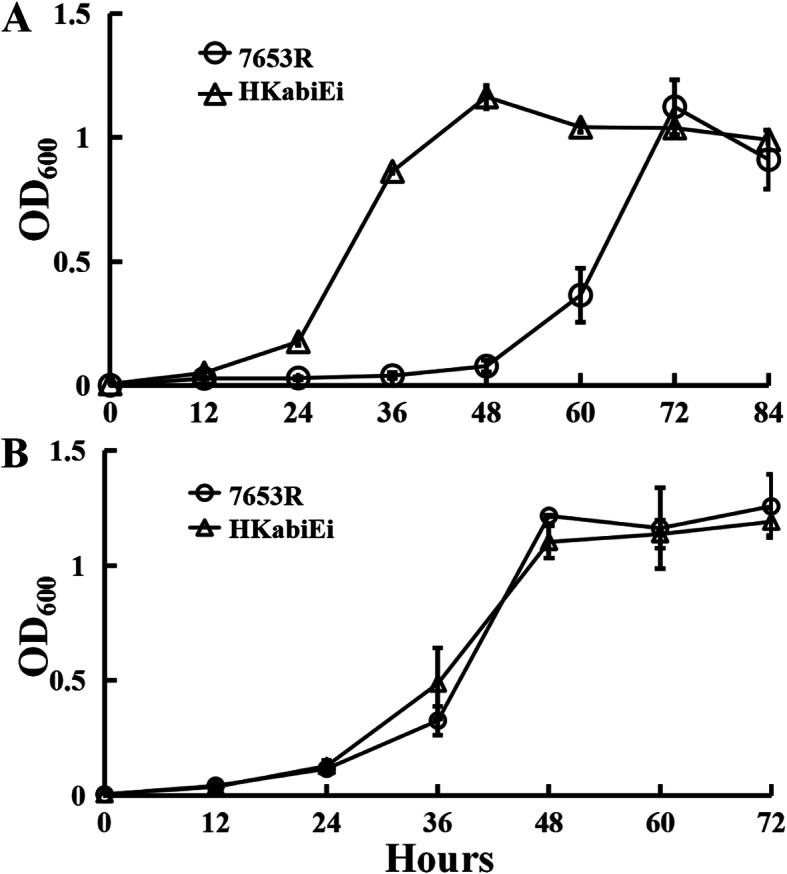
The effect of antibiotics on bacterial growth. Wild-type 7653R and mutant HKabiEi were grown in AMS Glc/NH4^+^ supplemented with 2 μg ml^−1^ Gm (**A**) or Cm (**B**). Data are from three biological samples plus and minus the standard error of the mean (± SEM)

It has been reported that TA systems constitute a native survival strategy of pathogenic bacteria and thus are potential targets of antibiotic drugs [19]. To examine the possibility that *abiEi* affects the antibiotic-resistance of *M. huakuii*, sensitivity to gentamicin (Gm) and chloramphenicol (Cm) at low concentration was assayed in AMS minimal medium. In the presence of 2 μg mL^− 1^ Gm, the mutant HKabiEi was grown to the early logarithmic phase at 24 h, and entered the stationary at 48 h postinoculation, whereas the parent strain 7653R was grown to the early logarithmic phase at 48 h, and entered the stationary phase at 72 h postinoculation (Fig. 3A). The resistance of the *M. huakuii* strain to 2 μg mL^− 1^ Cm stress was also estimated, the *abiEi* mutant HKabiEi showed no difference in growth as compared with the wild-type (Fig. 3B). These data indicated that mutantion in *abiEi* gene can display different antibiotic susceptibilities of *M. huakuii.*

### Role of the *abiEi* gene in the regulation of redox homeostasis

**Fig. 4:**
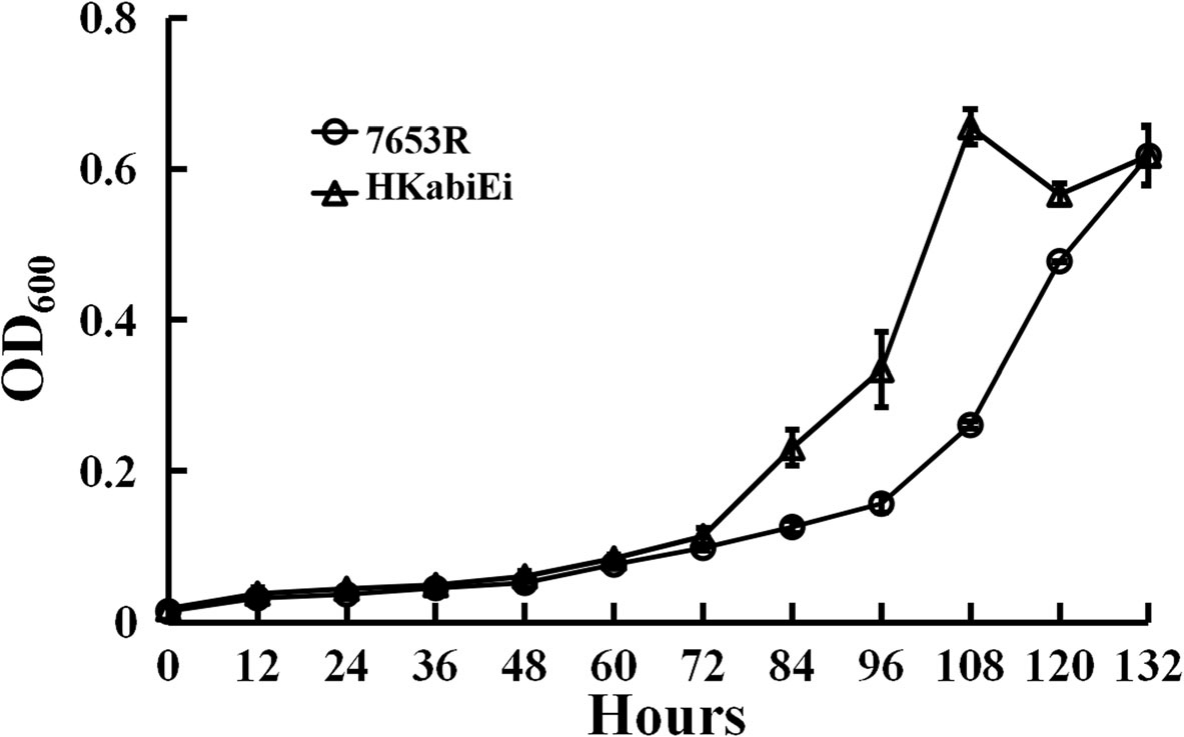
The effect of SNP on bacterial growth. Wild-type 7653R and mutant HKabiEi were grown in AMS Glc/NH4^+^ supplemented with 10 μg ml^− 1^ SNP. Data are from three biological samples plus and minus the standard error of the mean (± SEM)

It has been reported that the bacterial TA system is a module that plays a role in cell survival under stress conditions [20]. To determine the function of the *M. huakuii abiEi* gene in the regulation of redox homeostasis, the sensitivity of the *abiEi* mutant strain to the inhibition of growth by H_2_O_2_ or SNP, which could be due to changes in the cellular redox status [21], was examined. The zone of inhibition induced by H_2_O_2_ for mutant HKabiEi significantly decreased as compared with the wild-type 7653R strain (Table 1, Additional file 1). When *abiEi* on plasmid pBBR1MCS-5 was introduced into mutant HKabiEi, the resulting strain HKabiEi(pBBRabiEi) could rescue the variation of the inhibition zone, and showed hypersensitive to H_2_O_2_ as it made significantly bigger (*p* < 0.05) inhibition zone than the wild-type 7653R strain, whereas HKabiEi harboring an empty plasmid showed no significant difference with regard to mutant strain (Table 1, Additional file 1). The resistant to SNP stress was also estimated, and mutant HKabiEi was more resistant to 10 μg mL^− 1^ SNP compared with the wild-type 7653R strain (Fig. 4). These results indicated that *abiEi* gene plays a negative regulatory role in the oxidative stress response.

**Table 1:** Tolerance of strains to different concentrations of H_2_O_2_

Strains	Diameter(cm) c(H_2_O_2_)/(mmol·L^− 1^)		
	20	100	250
7653R	1.82 ± 0.08^a^	3.03 ± 0.09^a^	3.88 ± 0.18^a^
HKabiEi	1.64 ± 0.09^b^	2.60 ± 0.05^b^	3.43 ± 0.08^b^
HKabiEi(pBBRabiEi)	2.12 ± 0.27^c^	3.83 ± 0.12^c^	4.28 ± 0.52^c^
HKabiEi(pBBR1MCS-5)	1.55 ± 0.55^b^	2.70 ± 0.30^b^	3.45 ± 0.05^b^

The data are the average of at least three replicates. ^a, b, c^ values in each column followed by the same letter are not significantly different (*P* ≤ 0.05)

### Effect of mutation of *abiEi* on rhizosphere competition

**Fig. 5:**
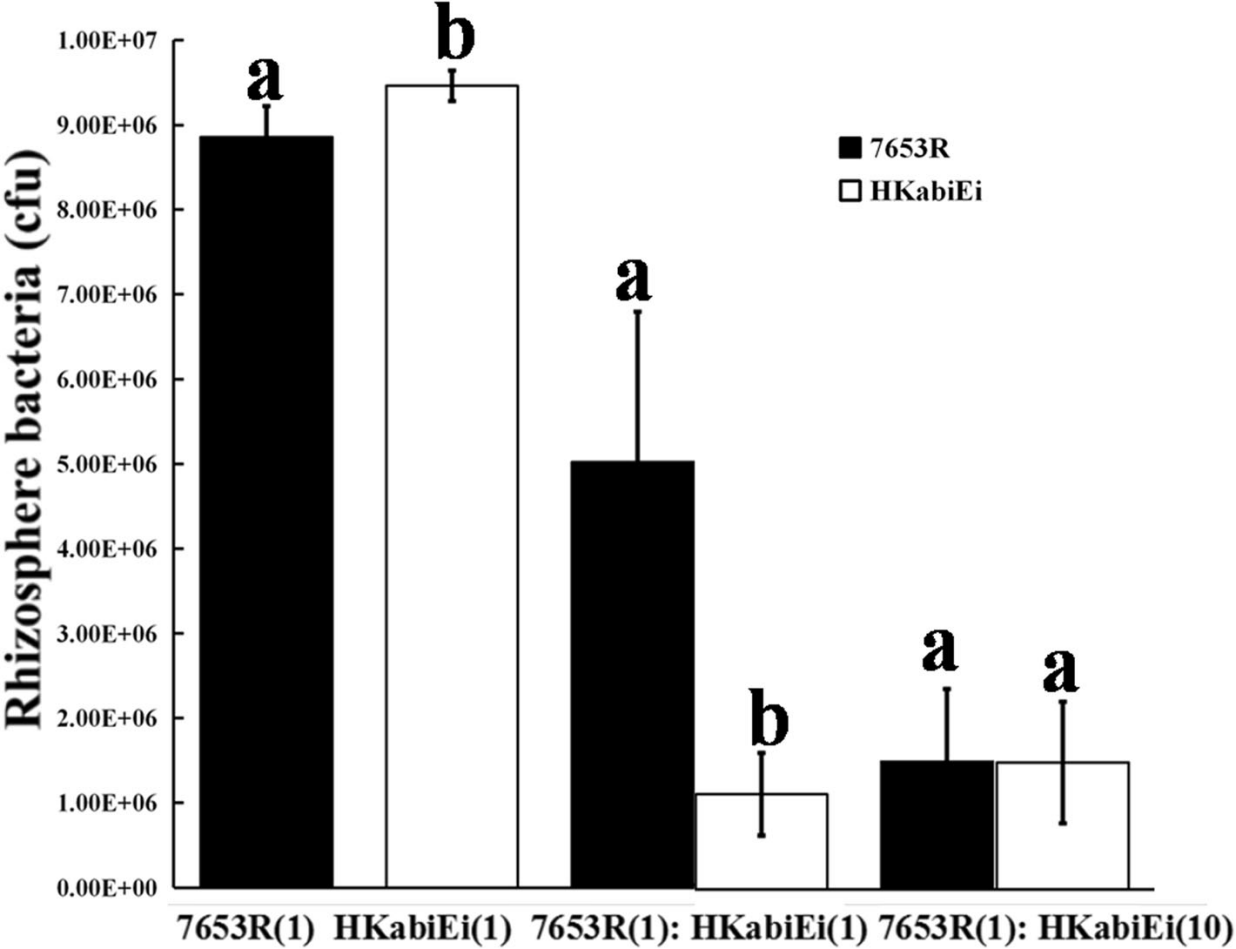
Bacteria recovered (7 dpi) from the rhizosphere of *Astragalus sinicus* plants following inoculation with wild-type 7653R and HKabiEi, both individually and together. Inoculation ratios are given on the *x* axis, with 1 corresponding to 10^3^ CFU. Number of bacteria (per plant) recovered from at least 5 plants (mean ± SEM) are shown. ^a,b^ Values with different letters are signifificantly different between mutant and wild-type control (two-way ANOVA, *P* < 0.05)

Competition between the *abiEi* mutant HKabiEi and parent strain 7653R for growth in the plant rhizosphere was measured by inoculating a low number of *M. huakuii* strains into the *A. sinicus* rhizosphere (10^3^ to 10^4^ CFU per seedling), and determining the total amount of bacteria after 7 days. When the mutant HKabiEi and the parent 7653R were inoculated alone into short-term colonization of sterile plant rhizosphere, the population density of the mutant strain was a little (but significantly) higher than that of wild-type (Fig. 5). When these strains were inoculated in equal proportions, mutant HKabiEi was at a significant disadvantage (32.90% of bacteria recovered). When strain HKabiEi was inoculated at a 10-fold excess over wild-type 7653R, it still accounted for only 48.68% of bacteria recovered (*t-*test; *P* ≤ 0.01). TA systems are ubiquitous bacterial systems that may function in metabolic stress management, but are also thought to play a role in virulence by helping pathogens survive stress [22]. The decreased ability of the *abiEi* mutant to complete in host plant rhizosphere shows that transcriptional regulator AbiEi is essential for colonization of the host plant rhizosphere by *M. huakuii*.

### Effect of mutation of *abiEi* on nodulation

**Fig. 6:**
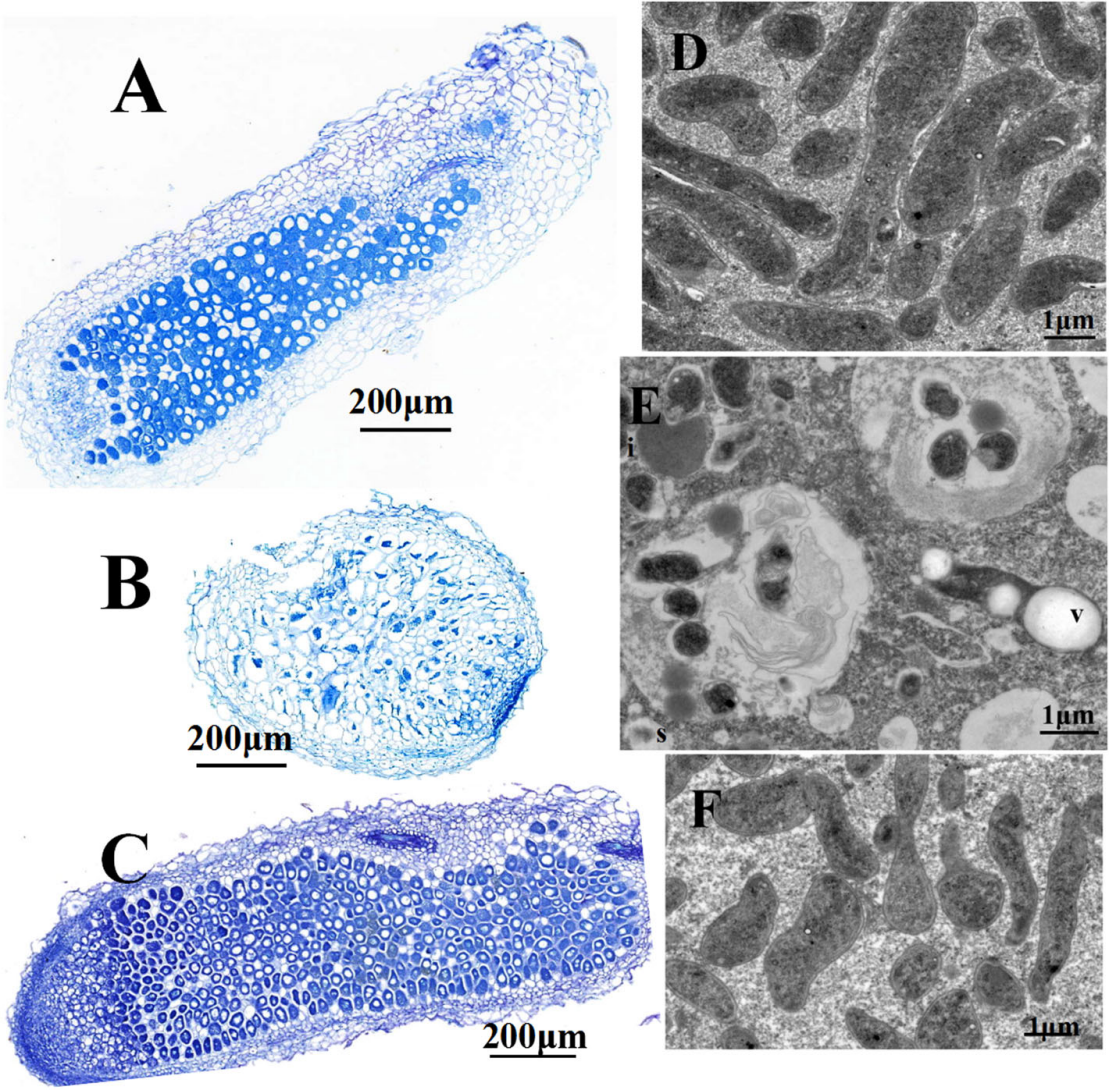
Structure of 4-week-old *Astragalus sinicus* nodules and bacteroids. Nodules were induced by *M. huakuii* 7653R (**A**, **D**), HKabiEi (**B**, **E**), HKabiEi(pBBRabiEi) (**C**, **F**). Scale bars = 200 μm (**A**, **B**, **C**), 2 μm (**D**, **E**, **F**). i, incrassated membrane; dissociation; v, vacuolation; s, senescing bacteroid

In order to assess the nodulation and nitrogen fixing capacity of the *abiEi* mutant, *A. sinicus* seedlings were inoculated with *M. huakuii* strains. Four weeks post-inoculation, the number, structure, and acetylene reduction activity (ARA) values of the nodules were analyzed (Table 2 and Fig. 6). No statistically significant difference in the number of nodules per plant was observed between plants inoculated with strain HKabiEi and plants inoculated with wild-type 7653R (Table 2). In contrast, the *abiEi* mutant HKabiEi elicited small white nodules on *A. sinicus*, and the plants inoculated with the strain HKabiEi were similar to that of plants without inoculation. A notable feature of our study was that the nitrogen fixation capacity was severely affected in the *abiEi* mutant HKabiEi, with a reduction from 35.20 nmol of C_2_H_4_ plant^− 1^ h^− 1^ in 7653R to 0.90 nmol of C_2_H_4_ plant^− 1^ h^− 1^ in the *abiEi* mutant (Table 2). Plants nodulated with the complemented strain HKabiEi(pBBRabiEi) had somewhat wild-type properties; approximately the same plant fresh weight, red nodules and reduce acetylene per nodule at the same rate as 7653R-inoculated.

**Table 2:** Symbiotic phenotype of 7653R and HKabiEi ^α,β^

Strain *M. huakuii*	Plant fresh weight (mg of plant)	Number of total nodules per plant	Acetylene reduction activity (nmol of ethylene/plant/h)	Acetylene reduction activity (nmol of ethylene/nodule/h)
7653R	106.05 ± 8.59^a^	17.50 ± 2.12^a^	35.20 ± 3.68 ^a^	2.01 ± 0.034 ^a^
HKabiEi	63.58 ± 3.94^b^	15.40 ± 4.39^a^	0.90 ± 0.12 ^b^	0.06 ± 0.07 ^b^
HKabiEi(pBBRabiEi)	93.58 ± 0.83^a^	7.00 ± 1.00^b^	15.03 ± 0.42 ^c^	2.17 ± 0.66 ^a^
Control ^γ^	54.84 ± .10^b^	0	0	0

^α^ Data are the average of at least 5 replicates. Acetylene reduction activity of nodules induced by *abiE* mutant strain HKabiEi or complementary strain HKabiEi(pBBRabiEi) was compared to that of nodules induced by the wild-type strain 7653R

^β a,b^ Values in each column followed by the same letter are not significantly different (*P* ≤ 0.05)

^γ^ Control: plants not inoculated with rhizobial strain

To further investigate the symbiotic role of *abiEi* in more detail, sections of mature nodules were prepared and examined by an Olympus microscope and scanning electron microscopy (SEM). Microscopic analysis of HKabiEi nodules showed that they were small and spherical instead of large and elongated like the wild-type, and compared with wild-type nodules, relatively few nodule cells were infected in the mutant nodules (Fig. 6). SEM analysis demonstrated an aberrant shape of *abiEi* bacteroids, which were reduced compared to wild-type bacteroids (Fig. 6). A quantitative analysis of bacteroid lengths demonstrated that the mean length of wild-type bacteroids was more than three times those of the *abiEi* mutant bacteroids, but not different from those of the complemented strains HKabiEi(pBBRabiEi). Moreover, the mutant infected nodule cells contained vacuolation and a small number of abnormal bacteroids, with incrassated membrane, scarce content in bacteroids and signs of premature senescence of endosymbiotic bacteria. The results suggested that the mutant-induced nodules were functionally defective.

### Activation of *abiEi* gene in 7653R-inoculated nodules

**Fig. 7:**
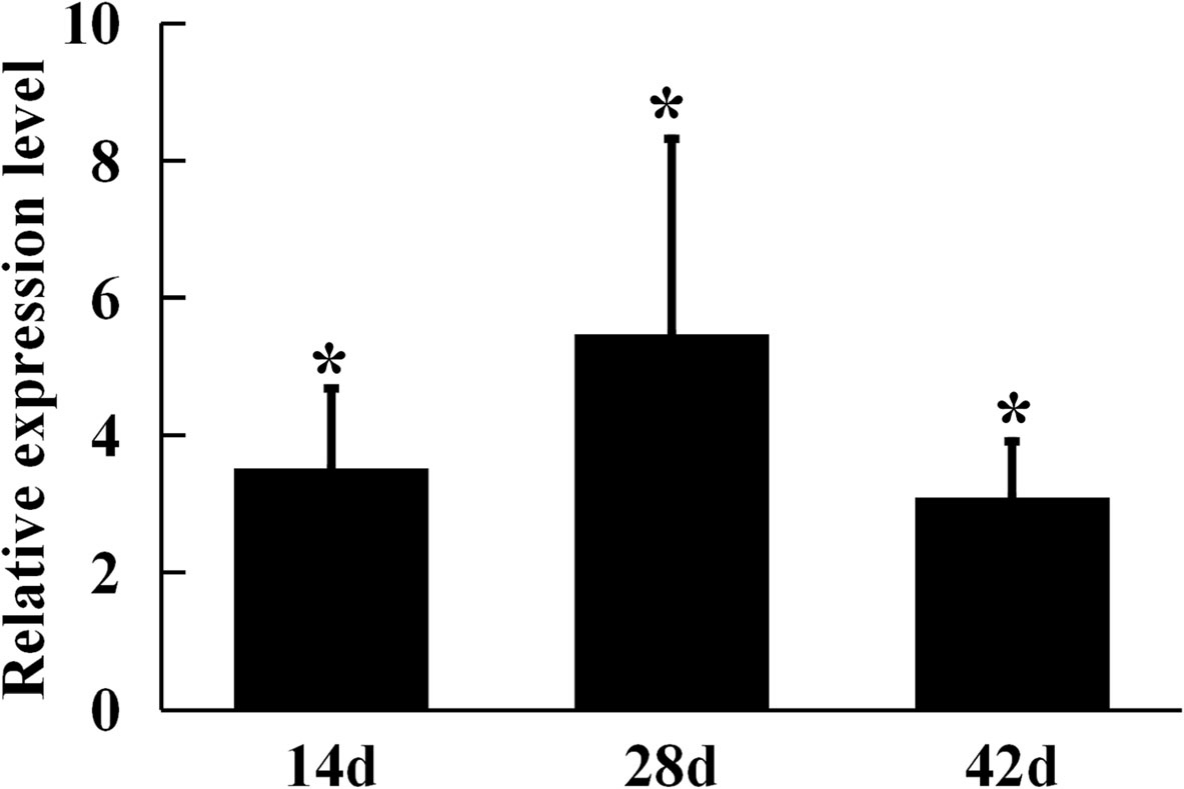
Expression patterns of *abiEi* gene in symbiotic nodules. Gene expression levels were examined by real-time RT-PCR. Nodules were collected on different days after inoculation with *M. huakuii* 7653R. Relative expression of *abiEi* gene involved in nodule bacteroids compared with 7653R cells growth in AMS medium. Data are the average of three independent biological samples (each with three technical replicates). Asterisk (*) indicates a significant difference (FC > 2, *P* < 0.05)

As toxin–antitoxin transcriptional regulator AbiEi plays a pivotal role in symbiotic nitrogen fixation, the expression of *abiEi* gene in root nodules was analyzed by quantitative RT-PCR (qRT-PCR). The *abiEi* gene expression was significantly up-regulated in the early stage of nodule formation (14 d), the nodule maturation stage (28 d) and the late stage (42 d) of nodule development and senescence, and the *abiEi* gene had the highest expression level (more than 5-fold) in nodules at 28 days post inoculation (Fig. 7). Therefore, *abiEi* gene expression was induced during the symbiotic interaction when compared with free-living cells growing in synthetic medium, and *abiEi* may play an important role in persistence of nodule bacteroids and prevention of premature nodule senescence.

### RNA-seq analyses of gene expression in the nodule bacteroids

RNA-Seq was performed to analyze the overall effect of *abiEi* on the transcription pattern of genes in *M. huakuii* bacteroids. *A. sinicus* root nodules induced by a *M. huakuii* wild-type or an *abiEi* gene mutant strain were collected at 28 dpi postinoculation. Total RNA was extracted, converted into cDNA libraries, and sequenced using Illumina paired-end sequencing technology. 52-million clean sequencing reads were gained from the RNA-seq transcriptomic analysis of the two samples, with an average of 26-million reads per sample. In total, 6590 expressed genes were detected by RNA-seq during the symbiosis between the strains and the *A. sinicus*. Gene expression level comparison analysis found that 116 genes were differentially expressed (*p*-value≤0.001, with log_2_ (FC) ≥ 3 and ≤ − 3), of which 5 were up-regulated and 111 were down-regulated (Tables 3 and 4). We validated the RNA-seq results by RT-PCR using several down-regulated genes as representatives. The datas showed that their expression was significantly decreased in mutant 28-day-old nodules compared to wildtype, which is consistent with the RNA-Seq data (Table 4). Among these differentially expressed genes, 23 (19.8%) were located on the chromosome, 54 (46.6%) were located on the plasmid pMHa, and 39 (33.6%) were located on the symbiotic plasmid pMHb, which contains nodulation genes (*nod*) as well as genes involved in nitrogen fixation (*nif* and *fix*). It is worth mentioning that all of the up-regulated genes were located on the chromosome, while more than 80% of the down-regulated genes were located on the extrachromosomal plasmids.

**Table 3:** List of 5 genes that showed significantly increased expression in *Astragalus sinicus* nodules, induced by the *abiEi* mutant strain compared to the wild-type 7653R

Gene ID	Location	Description	FC ^α^	*P* value
MCHK_RS00055	Chro	type IVb pilin	3.03	2.75E-05
MCHK_RS17855	Chro	flavin mononucleotide transferase	3.29	3.71E-08
MCHK_RS11320	Chro	hypothetical protein	3.53	2.43E-37
MCHK_RS11325	Chro	cytochrome b561	3.84	7.20E-27
MCHK_RS18405	Chro	hypothetical protein	4.45	4.88E-52

^α^ log_2_ of the fold change (FC) in expression of *A. sinicus* nodules induced by an *abiE* mutant versus the wild-type 7653R. *Chro* Chromosome

**Table 4:** List of 111 genes that showed significantly decreased expression in *Astragalus sinicus* nodules, induced by the *abiEi* mutant strain compared to the wild-type 7653R

Gene ID	Location	Description	FC ^α^	RT-PCR
Symbiotic nitrogen Fixation and nitrogen mechanism				
MCHK_RS32450	pMHb	electron transfer flavoprotein FixA	−8.38	− 9.98
MCHK_RS32495	pMHb	nitrogen fixation protein NifZ	−8.37	
MCHK_RS32500	pMHb	nitrogen fixation protein NifT	−8.64	
MCHK_RS32360	pMHb	nitrogen fixation protein NifQ	−7.12	
MCHK_RS31365	pMHa	Nif11-like leader peptide family natural product precursor	−6.00	
MCHK_RS31560	pMHa	Nif11 family protein	−4.35	
MCHK_RS32300	pMHb	nitrogen fixation protein NifX	−4.30	
MCHK_RS32485	pMHb	nitrogenase cofactor biosynthesis protein NifB	−4.53	
MCHK_RS32590	pMHb	alanine racemase	−4.28	
MCHK_RS32615	pMHb	aminotransferase	−4.82	
MCHK_RS34850	pMHb	pilus assembly lipoprotein	−3.11	
MCHK_RS31565	pMHa	amino acid epimerase	−4.46	
MCHK_RS31130	pMHa	N-acetyltransferase	−3.22	
MCHK_RS08325	Chro	ABC transporter permease	−4.28	
MCHK_RS11590	Chro	urea ABC transporter substrate-binding protein	−3.40	
MCHK_RS34620	pMHa	ABC transporter permease	−3.24	
MCHK_RS08320	Chro	ABC transporter permease	−3.16	
MCHK_RS08335	Chro	ABC transporter ATP-binding protein	−5.74	
MCHK_RS08330	Chro	ABC transporter substrate-binding protein	−5.15	
MCHK_RS07300	Chro	ABC transporter substrate-binding protein	−3.12	
MCHK_RS11580	Chro	urea ABC transporter permease subunit UrtC	−3.11	
Type VI secretion system				
MCHK_RS31520	pMHa	type VI secretion system baseplate subunit TssG	−6.17	
MCHK_RS31510	pMHa	type VI secretion system baseplate subunit TssE	−6.12	
MCHK_RS31500	pMHa	type VI secretion system contractile sheath large subunit	−6.08	
MCHK_RS31530	pMHa	type VI secretion system tip protein VgrG	−6.78	
MCHK_RS31415	pMHa	type VI secretion system baseplate subunit TssK	−5.84	
MCHK_RS31515	pMHa	type VI secretion system baseplate subunit TssF	−7.52	
MCHK_RS31420	pMHa	type VI secretion system-associated FHA domain protein TagH	−5.17	
MCHK_RS31490	pMHa	type VI secretion system protein TssA	−4.94	
MCHK_RS31495	pMHa	type VI secretion system contractile sheath small subunit	−3.14	
MCHK_RS31505	pMHa	type VI secretion system tube protein Hcp	−6.97	
MCHK_RS31430	pMHa	type VI secretion system membrane subunit TssM	−6.87	
MCHK_RS31425	pMHa	type IV/VI secretion system protein	−5.73	
Electron transport and antioxidant system				
MCHK_RS34955	pMHa	ferredoxin	−5.68	
MCHK_RS32340	pMHb	cytochrome P450	−10.19	
MCHK_RS32465	pMHb	ferredoxin family protein	−8.26	−9.60
MCHK_RS32535	pMHb	cytochrome P450	−5.40	
MCHK_RS32600	pMHb	4Fe4S-binding protein	−3.77	
MCHK_RS31355	pMHa	peroxiredoxin	−8.09	
MCHK_RS31120	pMHa	FAD-dependent oxidoreductase	−3.91	
MCHK_RS32355	pMHb	flavin-dependent oxidoreductase	−8.59	
MCHK_RS34760	pMHa	cold-shock protein	−6.69	
MCHK_RS32350	pMHb	short-chain dehydrogenase	−8.59	
MCHK_RS34755	pMHa	cysteine protease avirulence protein AvrRpt2	−9.06	
MCHK_RS31555	pMHa	L-ascorbate oxidase	−9.55	−13.04
MCHK_RS08620	Chro	FAD-binding oxidoreductase	−3.14	
MCHK_RS32460	pMHb	FAD-dependent oxidoreductase	−6.41	
MCHK_RS32390	pMHb	cold-shock protein	−6.31	
MCHK_RS31275	pMHa	NADPH-dependent F420 reductase	−7.42	
Nucleic acid metabolism				
MCHK_RS31350	pMHa	recombinase family protein	−3.09	
MCHK_RS31480	pMHa	DNA-binding response regulator	−4.52	
MCHK_RS32415	pMHb	integrase	−4.71	
MCHK_RS31865	pMHb	transposase	−4.70	
MCHK_RS34785	pMHa	IS110 family transposase	−7.61	
MCHK_RS31270	pMHa	IS6 family transposase	−7.60	
MCHK_RS31860	pMHb	IS6 family transposase	−4.07	
MCHK_RS33710	Chro	IS6 family transposase	−3.65	
MCHK_RS34835	pMHa	IS6 family transposase	−3.60	
MCHK_RS32210	pMHb	IS6 family transposase	−4.68	
MCHK_RS34745	pMHa	IS6 family transposase	−4.63	
MCHK_RS31375	pMHa	IS6 family transposase	−3.26	
MCHK_RS31385	pMHa	IS21 family transposase	−6.61	
MCHK_RS05160	Chro	translocated repetitive protein	−8.36	
MCHK_RS31125	pMHa	maturation protein 1	−3.03	
MCHK_RS32635	pMHb	autoinducer synthase	−5.04	
MCHK_RS31360	pMHa	RNA polymerase sigma-54 factor	−7.22	
MCHK_RS31390	pMHa	GntR family transcriptional regulator	−6.74	
MCHK_RS32640	pMHb	LuxR family transcriptional regulator	−6.49	
MCHK_RS31250	pMHa	XRE family transcriptional regulator	−5.93	
MCHK_RS34740	pMHa	hypothetical protein(transcriptional regulator)	−7.35	
MCHK_RS32365	pMHb	RNA polymerase sigma-54 factor	−5.62	
MCHK_RS34845	pMHa	DNA-binding response regulator	−3.47	
MCHK_RS31380	pMHa	nuclear transport factor 2 family protein	−8.90	
MCHK_RS32345	pMHb	nuclear transport factor 2 family protein	−8.59	− 8.83
MCHK_RS34720	pMHa	DDE-type integrase/transposase/recombinas	−6.91	
MCHK_RS31330	pMHa	(Nucleotidyl transferase proteins) NTP transferase	−9.47	−4.57
Carbon mechanism				
MCHK_RS17040	Chro	formyl transferase	−4.39	
MCHK_RS17020	Chro	glycosyltransferase	−4.37	
MCHK_RS00340	Chro	acyltransferase	−3.88	
MCHK_RS08380	Chro	acyltransferase	−3.19	
MCHK_RS31550	pMHa	tyrosinase	−8.35	
MCHK_RS32545	pMHb	metalloenzyme	−3.22	
MCHK_RS17025	Chro	glycosyl hydrolase	−3.70	
MCHK_RS32620	pMHb	alkaline phosphatase	−4.31	
MCHK_RS32605	pMHb	transketolase	−3.46	
MCHK_RS31340	pMHa	EamA family transporter	−9.09	−6.79
MCHK_RS31315	pMHa	EamA family transporter	−5.96	
Hypothetical protein				
MCHK_RS34775	pMHa	hypothetical protein	−9.07	
MCHK_RS32295	pMHb	hypothetical protein	−9.03	
MCHK_RS33605	pMHb	hypothetical protein	−7.97	
MCHK_RS31545	pMHa	hypothetical protein	−7.62	
MCHK_RS31320	pMHa	hypothetical protein	−9.16	
MCHK_RS34780	pMHa	hypothetical protein	−6.56	
MCHK_RS09340	Chro	hypothetical protein	−6.25	
MCHK_RS32420	pMHb	hypothetical protein	−6.18	
MCHK_RS33530	Chro	hypothetical protein	−5.37	
MCHK_RS32630	pMHb	hypothetical protein	−5.22	
MCHK_RS32550	pMHb	hypothetical protein	−5.03	
MCHK_RS34750	pMHa	hypothetical protein	−4.98	
MCHK_RS32395	pMHb	hypothetical protein	−4.90	
MCHK_RS31405	pMHa	hypothetical protein	−4.55	
MCHK_RS31285	pMHa	hypothetical protein	−4.16	
MCHK_RS31535	pMHa	hypothetical protein	−4.12	
MCHK_RS34940	pMHb	hypothetical protein	−3.79	
MCHK_RS32575	pMHb	hypothetical protein	−3.59	
MCHK_RS09315	Chro	hypothetical protein	−3.52	
MCHK_RS31115	pMHa	hypothetical protein	−4.39	
MCHK_RS32680	pMHb	hypothetical protein	−3.38	
MCHK_RS32585	pMHb	hypothetical protein	−4.70	
MCHK_RS32595	pMHb	hypothetical protein	−4.96	
MCHK_RS31435	pMHa	hypothetical protein	−6.69	

^α^ log_2_ of the fold change (FC) in expression of *A. sinicus* nodules induced by an abiE mutant (*abiE* mt nod) versus the wild type 7653R (wt nod); GHMP kinase: the galacto*kinase*, homoserine kinase, mevalonate kinase, and phosphomevalonate kinase; *ROK* repressor: open reading frame, kinase; MFS: major facilitator superfamily

Among the five up-regulated genes, one encodes type IVb pilin, one codes FMN transferase, one encodes cytochrome b561, and two encode proteins of unknown function. To categorize these differences into modules of biological relevance, the 111 down-regulated genes were annotated. They were functionally classified into 6 categories, which were involved in symbiotic N_2_-fixation and nitrogen mechanism (*n* = 21, 18.9%), type VI secretion system (*n* = 12, 10.8%), electron transport and antioxidant system (*n* = 16, 9.4%), nucleic acid metabolism (*n* = 27, 24.3%), Carbon mechanism (*n* = 11, 9.9%), and hypothetical protein (*n* = 16, 21.6%) (Table 4). In particular, one *fix* gene and seven *nif* genes were required for induction of nitrogen-fixing nodules on *A. sinicus,* and T6SS is rhizobial protein injection machinery with a positive role in Rhizobium-legume symbiosis [23]. The number of differentially expressed symbiosis-associated genes indicated that regulator AbiEi affects the transcription of a wide range of genes involved in the legume–rhizobium interactions. Further analysis of the differentially expressed genes identified a subset involved in electron transport and antioxidant system. The number of affected the expression of genes involved in the electron transport chain and antioxidant responses also suggested that regulator AbiEi plays an important role in antioxidant systems and the regulation of the electron transport chain. Moreover, 12 genes of T6SS were also found to be significantly over-represented. The genome of many rhizobia encodes T6SS but their role in symbiosis is mostly unknown. However, a functional T6SS, with an effect on symbiosis, has only been shown in *R. leguminosarum* [24]. Furthermore, 27 genes associated with nucleic acid metabolism were found among the 111 genes showing decreased expression in mutant nodules: 10 coding for transposases, six coding for transcriptional or response regulator. Previous studies suggested that transposases (or insertion sequence), transcriptional or response regulator are linked with functions to rhizobial nodulation [25–29]. In addition, 10 carbon mechanism genes and 24 genes of unknown function were found to be significantly down-regulated in the mutant nodules. qRT-PCR was further performed to confirm changes in gene expression determined by RNA-seq. Seven down-regulated genes in five different functional categories were significantly less in HKabiEi-induced nodules compared to nodules induced by wild-type. These results are largely consistent with the changes seen in the RNA-seq assay results.

## Discussion

The type IV TA system AbiE was recently reported, and belonged to the poorly characterized but widespread abortive infection/TA family [12]. To date, there is almost no information of rhizobial AbiE TA system. The *Mesorhizobium huakuii* AbiE system consists of toxin AbiEii and antitoxin AbiEi. Here we examine the antitoxin AbiEi, which is essential for transcriptional repression of the abiE operon. To investigate the mode of action of this antitoxin, we focus on a *abiEi* mutant strain of *M. huakuii* that is affected with regard to its symbiotic capacity and stress response.

TA systems are key regulators of bacterial persistence, and are linked to many roles in cell physiology, such as plasmid maintenance, stress response, antibiotic resistance, virulence and programmed cell death [30]. Our experiments indicated that the *M. huakuii* AbiE system can work as a functional TA module in the growth, stress response and antibiotic resistance. Firstly, the *abiEi* mutant showed slight growth inhibition effect when the strains were cultured in nutrient-rich medium. Due to lack of the antitoxin AbiEi, the *abiEi* mutant can not neutralize the toxin’s activity, and therefore, causes growth stasis. This finding is in agreement with two previous studies reporting that overexpression of the toxin gene in *Escherichia coli* inhibited its growth [13, 31]. Interestingly, the *abiEi* mutant showed no significant difference in AMS minimal medium, suggesting that AbiE TA system does not perform growth inhibition when strains grow at low cell density in nutrient-limiting minimal medium. Secondly, the *abiEi* mutant displayed significant difference on susceptibility of antibiotics tested. Mutantion in *abiEi* did not affect *M. huakuii* chloramphenicol resistance, but displayed an enhanced capacity of *M. huakuii* to gentamicin resistance [32]. It has been reported that the activity of TA system may contribute to the maintenance of antibiotic resistance. Thirdly, compared to wild-type, the *abiEi* mutant showed enhanced resistance to the oxidizing and reducing agents, H_2_O_2_ and SNP. It was recently reported that the production of Reactive Oxygen Species (ROS) is a common mechanism of cell death induced by bactericidal antibiotics [33].

The symbiotic performance of the *abiEi* mutant was altered at large levels of nodule function and maintenance. We have observed that the *M. huakuii abiEi* mutant induced the same number of nodules, exhibited a dramatical decrease in the nitrogen-fixing activity of root nodules (reduced by approx. 97%). In fact, a *S. meliloti* strain with a mutant antitoxin gene also had a reduced nitrogen-fixing activity, but formed a higher number of root nodules, which suggested that the host plant was starved for nitrogen due to the inefficient nitrogen fixation, thus initiation of additional nodules was permitted on the roots [10]. The reason why the *M. huakuii abiEi* mutant did not form more nodules may be due to decreased competitive ability. The root nodules induced by antitoxin gene mutant had decreased size, small amounts of infected cells and bacteroid content, and a severe senescent phenotype. These results indicated that inactive antitoxin AbiEi may affect the formation of the symbiotic nodules on the host roots. In fact, it has been reported that mutant in a functional toxin NtrR increased the symbiotic efficiency of *S. meliloti* [18], whereas a *S. meliloti* strain with a mutant antitoxin gene had a reduced nitrogen-fixing activity [10]. This symbiotic defect of the antitoxin AbiEi insertion mutant can be explained as a result of the disruption of balance between toxin and antitoxin. The *abiEi* mutant was defective in transcriptional repression of the *abiE* operon, and therefore, overexpressed the toxin AbiEii. On the one hand, many of toxins may induce programmed cell death by directly interacting with bacteroids [34]. Actually, the antitoxin was required for maintenance of transcriptional repression throughout nodule development as its expression was consistently significantly up-regulated in the nodules. On the other hand, a toxin VapC from the Leptospiral VapBC toxin-antitoxin module was reported to display ribonuclease activity on the initiator tRNA [35]. As a result, overexpressing the toxin AbiEii may result in a high percentage of down-regulated genes such as those which are functionally symbiosis related (Table 4**)**.

Previous reports indicated that the Toxin-Antitoxin is a posttranscriptional regulator of metabolic flux, and antitoxin functions as a transcriptional autoregulator [36, 37]. Henceforth, a RNA-seq experiment was performed to compare the transcript profiles between the root bacteroids infected by *abiEi* mutant and wild-type 7653R. Firstly, lack of *M. sativa* antitoxin protein has been reported to continuously decrease expression of *nif* genes in the nodules [10], similar result also happened in the *abiEi* mutant. Lack of the antitoxin AbiEi reduced transcription of 8 nitrogen fixation genes and 13 genes related to nitrogen transport and mechanism. These suggest that antitoxin AbiEi plays a crucial role in legume-rhizobial symbiosis by maintaining the level of nitrogen assimilation and mechanism activity. Secondly, Lack of the antitoxin AbiEi reduced transcription of T6SS, which comprises a series of proteins with structural homology to bacteriophage tail proteins and membrane proteins [38]. It has been reported that T6SS plays a major role in mediating interbacterial competition and might contribute to virulence in plant pathogenic bacteria [39], and *Rhizobium etli* mutants affected in T6SS structural genes produced plants with lower dry weight and smaller nodules [23]. Thirdly, mutation in AbiEi decreased the activity of redox enzymes such as peroxiredoxin, oxidoreductase, dehydrogenase, oxidase and reductase. Those enzymes were able to effectively reduced intracellular ROS production and involved in redox balance and respiration [40]. It has been reported that antioxidant proteins are critical for nodule activity, and a better antioxidant metabolism can lead to delayed senescence of nodules [41–43]. Fourthly, five down-regulation genes are involved in electron transfer. It has been reported that nitric oxide (NO) was also found to play a metabolic role in nodule energy metabolism, and electron transfer chains were found to significantly contribute to NO production in N_2_-fixing nodules [44]. Fifthly, inactivation of the AbiEi response regulator directly reduced the activation of four transcriptional regulators (*MCHK_RS31390*, *MCHK_RS32640*, *MCHK_RS31250* and *MCHK_RS34740*) and two response regulators (MCHK_RS31480 and MCHK_RS34845). Previous studies suggested that many transcriptional or response regulators are linked with functions to rhizobial nodulation [25–28]. Taken together, *M. huakuii* AbiEi plays an important role in root nodule symbiosis by regulation of nitrogen fixation gene expression, interbacterial competition, redox balance and respiration, bacteroid formation and senescence.

## Conclusions

Bacterial Toxin-antitoxin systems are composed of bicistronic operons encoding a stable toxin that can harm the host cell and its cognate labile antitoxin. The contribution of toxin-antitoxin to symbiosis and stress response was investigated using the *M. huakuii abiEi* mutant. The results showed the *abiEi* mutant strain displayed decreased antioxidative capacity and enhanced gentamicin resistance, and was severely impaired in symbiotic nitrogen-fixing capacity. A quantitative RNA-Seq based transcriptomics approach was also applied to reveal the global transcriptomic responses to AbiEi defect in *M. huakuii* bacteroids from *A. sinicus* root nodules. Compared to the 7653R bacteroids, there were 5 genes were up-regulated and 111 genes were down-regulated in HKabiEi bacteroids. This study provided majority of these differentially expressed genes were grouped into 6 categories and a valuable insight into AbiEi-mediated mechanisms during *M. huakuii*-*A. sinicus* symbiosis. Furthermore, this study has generated an abundant list of transcript from *M. huakuii* which will provide a fundamental basis for future functional genomic research in *M. huakuii* and other closely related species.

## Methods

### Bacterial growth and media

The host plant is *Astragalus sinicus*, which is distributed in all provinces of the Yangtze River Basin in China and cultivated all over the country. The strains, plasmids and primers used in this study are listed in Table 5. *M. huakuii* strains were grown at 28 °C in either Tryptone Yeast extract (TY) [49] or Acid Minimal Salts medium (AMS) [50] with D-glucose (10 mM) as a carbon source and NH_4_Cl (10 mM) as a nitrogen source. For growth and qRT-PCR experiments, cells were grown in AMS. When required, the following antibiotics were used at the following final concentrations (μg mL^− 1^): Streptomycin (Str), 500; Ampicillin (Amp), 50; Kanamycin (Km), 20, or 50 (for *E. coli* growth); Neomycin (Neo), 80, or 250 (for making *abiEi* mutant); Gentamicin (Gm), 20; Spectinomycin (Spe), 100; Tetracycline (Tc), 5. To monitor culture growth, strains were grown at 28 °C with shaking (200 rpm) in liquid AMS or TY, and culture optical density at 600 nm (OD_600_) was measured during the culture period. For antibiotic and sodium nitroprusside (SNP) sensitivity assay, gentamicin, chloramphenicol and SNP were added to each AMS medium at a final concentration of 2 μg mL^− 1^, 2 μgmL^− 1^, and 10 μg mL^− 1^, respectively.

**Table 5:** Strains, plasmids and primers used in this experiment

Strains	Description	Reference, Source, Sequence
*M. huakii* 7653R	Wild type, Nod^+^ on *Astragalus sinicus*	[45]
HKabiEi	7653R *abiEi*:pk19mob, Str^r^ Neo^r^	This study
HKabiEi(pBBRabiEi)	Strain HKabiEi harboring plasmid pBBRabiEi	This study
HKabiEi(pBBR1MCS-5)	Strain HKabiEi harboring plasmid pBBR1MCS-5	This study
Plasmids		
pETAbiE	AbiE_F/AbiE_R PCR product in pET-28a(+), Km^r^	This study
pETAbiEii	AbiE_F/AbiEii_R PCR product in pET-28a(+), Km^r^	This study
pK19mob	pK19mob pUC19 derivative *lacZ mob* Km^r^	[46]
pRK2013	Helper plasmid for mobilizing plasmids Km^r^	[47]
pKabiEi	abiEiUP/abiEiLW PCR product in pK19mob, Km^r^	This study
pBBR1MCS-5	lacPOZ′ mob, broad host range, Gm^r^	[48]
pBBRabiEi	cabiEi_F/cabiEi_R PCR product in pBBR1MCS-5, Gm^r^	This study
Primer^**a**^		
AbiE_F	Sense primer used for AbiE system and *abiEii* gene expression	TTTGGATCCATGTCCTTGGTTGAACCCGA
AbiE_R	Antisense primer used for AbiE system expression	TTTAAGCTTGTGCAGGTCGTAGTAGTGGC
AbiEii_F	Antisense primer used for *MCHK_RS33185* (*abiEii)* expression	TTTGGATCCGTGAGCACCGACGCCTATCG
abiEiUP	Sense primer for *MCHK_RS33180* (*abiEi*) mutation	TTTAAGCTTGAACCCGACAGCGATCTCCG
abiEiLW	Antisense prime for *MCHK_RS33180* (*abiEi*) mutation	TTTTCTAGAACTTGGCCTCGCTCGATGAG
abiEipimap	Mapping PCR primer for *abiEi*	TGACTCCAGCGCAGCCGTCA
cabiEi_F	Sense primer for *abiEi* complementation	TTTAAGCTTGCCACCGACCTTTTATCCTG
cabiEi_R	Antisense primer for *abiEi* complementation	TTTTCTAGAGCCCTTGAACAGCAGCCGCG
pK19A	pK19mob mapping primer	ATCAGATCTTGATCCCCTGC
pK19B	pK19mob mapping primer	GCACGAGGGAGCTTCCAGGG
Q16SrRNAF	Sense primer for qRT-PCR of 16S rDNA	AACTGAGATGGCTTTTGGAG
Q16SrRNAR	Antisense primer for qRT-PCR of 16S rDNA	GGATGACGTCAAGTCCTCAT
Q32450F	Sense primer for qRT-PCR of *MCHK_RS*32450	TGGCGAGGTTACCGTACTCA
Q32450R	Antisense primer for qRT-PCR of *MCHK_RS*32450	AGCGTGTCTGAGCCGGCAAA
Q32465F	Sense primer for qRT-PCR of *MCHK_RS*32465	GCTCTACCAGAACCGCTATC
Q32465R	Antisense primer for qRT-PCR of *MCHK_RS*32465	TCGTACAACTCGTAGCATTT
Q32345F	Sense primer for qRT-PCR of *MCHK_RS*32345	CAACATCCATCAGATCACGA
Q32345R	Antisense primer for qRT-PCR of *MCHK_RS*32345	TGGCGCATTTCTCCGTCTTT
Q31505F	Sense primer for qRT-PCR of *MCHK_RS*31505	CAATGGAGTTTCTCAATGGA
Q31505R	Antisense primer for qRT-PCR of *MCHK_RS*31505	TATAGAGAAGCAGAGGCGTT
Q31555F	Sense primer for qRT-PCR of *MCHK_RS*31555	TGATCAGGCAGAACCACGGC
Q31555R	Antisense primer for qRT-PCR of *MCHK_RS*31555	ATCAAGGTCCGTACCCGTTA
Q31330F	Sense primer for qRT-PCR of *MCHK_RS*31330	TCGGCTCAGAATATCCTCGT
Q31330R	Antisense primer for qRT-PCR of *MCHK_RS*31330	CGAAATGAAGTCGCTTAGTA
Q31340F	Sense primer for qRT-PCR of *MCHK_RS*31340	ATTGCCAGCGCCAGCCAGGG
Q31340R	Antisense primer for qRT-PCR of *MCHK_RS*31340	GGCCGGGCTGCTCTATCTCG
QabiEiF	Sense primer for qRT-PCR of *abiEi*	TCAAGAGCTTCGCCGGATCG
QabiEiR	Antisense primer for qRT-PCR of *abiEi*	ACGGATCACGGCGCGATAGT
QabiEiiF	Sense primer for qRT-PCR of *abiEii*	GGCCCGCTCAATGAGTTCCT
QabiEiiR	Antisense primer for qRT-PCR of *abiEi*	TCTGATACCAAAGCAGCAGG

^a^Restriction sites in primer sequences are underlined

### Expression of AbiE system and AbiEii protein in *E. coli*

The AbiE system coding sequence of 1.42 kb was amplified by PCR from 7653R genomic DNA by using primers AbiE_F and AbiE_R, and the *AbiEii* coding sequence of 0.7 kb was amplified by using primers AbiE_F and AbiEii_R. PCR products were digested with *Bam*HI and *Hin*dIII and cloned into the pET-28a(+), and the resulting plasmids were designated pETAbiE for AbiE system and pETAbiEii for toxin gene *abiEii*. The recombinant plasmids were further transformed in expression host *E. coli* BL21(DE3). Transformants obtained were grown in LB supplemented with appropriate antibiotics. The cultures were induced at OD_600_ 0.4 with 1 mM isopropyl b-thiogalactopyranoside (IPTG) and samples were collected every hour for 6 h. The experiment was repeated three times.

### Construction and complementation of *abiEi* gene mutant strain of *M. huakuii* 7653R

A single-crossover integration mutation in *abiEi* was made in 7653R. Primers abiEiUP and abiEiLW were used to PCR amplify the *abiEi* region from 7653R genomic DNA, and the 650 bp internal fragment of the *abiEi* gene was cloned into the *Hind*III and *Xba*I sites of pK19mob, giving plasmid pKabiEi. Plasmid pKabiEi was transferred from *E. coli* to 7653R and recombined into the genomic *abiEi* region via single crossover to give strain HKabiEi. Insertions into the *abiEi* gene of strain 7653R were confirmed by colony PCR using the abiEimap primer and a pK19mob-specific primer pK19A or pK19B [45, 49].

To complement the *abiEi* mutant, primers cabiEi_F and cabiEi_R were used to amplify the complete *abiEi* gene from *M. huakuii* 7653R genomic DNA. The PCR product was digested with *Hind*III and *Xba*I and cloned into the broad-host-range vector pBBR1MCS-5, resulting in plasmid pBBRabiEi. Plasmid pBBRabiEi was mated into the mutant strain HKabiEi using the triparental mating method as previously described, while conjugation of HKabiEi with empty vector (pBBR1MCS-5) was used as negative control.

### Cellular sensitivity to H_2_O_2_

Rhizobial cultures were grown in TY medium up to an OD_600_ of 1, washed and resuspended in sterilized phosphate buffered saline (PBS) 1 × (136 mM NaCl, 2.6 mM KCl, 8.0 mM Na_2_HPO_4_, 1.5 mM KH_2_PO_4_). Antioxidation activity were determined by agar diffusion assay using filter paper disc diffusion method. Agar plates made from TY were spread with 100 μL of bacterial cultures containing 1 × 10^9^ cfu mL ^− 1^. Sterile and dried filter paper discs 6 mm in diameter, impregnated with different concentrations of H_2_O_2_, were placed on the TY media plate on which bacteria had been spread. Plates were incubated at 28 °C for 96 h. Results were determined by measuring the diameter of the zones of growth inhibition surrounding the disc. The presence of clear inhibition zones around the bacterial growth indicated the antimicrobial activity. The experiment was repeated at least three times to ensure the reproducibility of the results. The data were analyzed with two-way ANOVA (*P* < 0.05).

### Plant experiment and cytological study of nodules

*Astragalus sinicus L.* was used as a host plant to test nodulation of the *M. huakuii* strains. Seeds were surface-sterilized, placed in 500 mL pots at two seeds per pot filled with sterile black vermiculite containing nitrogen-free Fahraeus solution. Inoculation with a bacterial suspension of about 10^8^ cells/seed was performed on 7-day-old seedlings. The cultivation was carried out in a controlled environment chamber with 16 h light/8 h dark period (day/night temperature, 22 °C and 20 °C). Acetylene reduction rate per plant was determined at 31 days postinoculation (dpi) as previously described. The experiment consisted of two independent experiments, each of which had five repeats, and statistical differences were analyzed with one-way ANOVA (*P* < 0.05).

Nodules at 31 dpi were fixed for 12 h at 4 °C with 2.5% glutaraldehyde, rinsed, and post-fixed in 1.5% phosphate-buffered osmium tetroxide. Ultra-thin sections stained with lead citrate were examined using a Hitachi H-7100 transmission electron microscope. Sections were cut with a microtome and stained with toluidine blue for light microscopy.

### Rhizosphere colonization

Rhizosphere colonization was performed as described previously. *Astragalus sinicus* seedlings were germinated and grown for 7 days as described above for acetylene reduction activity, and inoculated with *M. huakuii* 7653R and HKabiEi in the cfu ratios 1000:0, 0:1000, 1000:1000 and 1000:10000. Shoots were cut-off after 7 days (14 days after plant), and 10 mL of sterile phosphate-buffered saline (PBS) buffer (pH 7.4) was added to the roots and vortexed for 15 mins. The samples were further serially diluted and plated on TY agar plates containing either streptomycin and neomycin (only HKabiEi will grow) or streptomycin (both 7653R and HKabiEi will grow), giving the total number of viable rhizosphere- and root-associated bacteria. The ratio of the number of mutant strains to total number of mutant and wild-type strains was calculated for each inoculation test. Each treatment consisted of 10 replications, each of which consisted of a single plant, and statistical differences were analyzed with one-way ANOVA (*P* < 0.05).

### RNA isolation and quantitative RT-PCR analysis

Quantitative real-time reverse transcription PCR (qRT-PCR) was performed to determine the *abiEi* gene expression level in *A. sinicus* nodules and validate the results of RNA-seq. The total RNA was isolated using Trizol reagent from free-living *M. huakuii* 7653R or mutant HKabiEi cultivated in AMS liquid medium, or root nodules which were harvested from *A. sinicus* inoculated with strain 7653R or mutant HKabiEi. RNA were reverse transcribed into cDNA using the SuperScript II reverse transcriptase and random hexamers. qRT-PCR analysis was performed using a SYBR Premix ExTaq kit following the manufacturer’s instructions on the BIO-RAD CFX96 Real-Time PCR Detection System. The primer sequences are shown in Table 5. The 16S rRNA gene of *M. huakuii* 7653R was used as a calibrator gene, and the data were obtained. Three independent biological replicates were included in the analysis, and the relative expression levels of the target genes were normalized using the 2^−ΔΔCT^ method.

### RNA-seq library preparation and sequencing using the illumina genome analyzer

At 4 weeks post-inoculation, the nodules of plants inoculated with HKabiEi or 7653R were harvested, immediately frozen in liquid nitrogen and stored at − 80 °C. Total cellular RNA was isolated from frozen nodule tissues using Trizol Reagent (Invitrogen) and RNeasy Mini Kit (Qiagen). Total RNA of each nodule sample was treated with RNase-free DnaseI (TAKARA, Dalian, China), and the absence of contaminating DNA was confirmed by PCR-based assays. Total RNA was assessed using Agilent 2100 Bioanalyzer (Agilent Technologies, Palo Alto, CA, USA), and NanoDrop (Thermo Fisher Scientific Inc). 1 μg total RNA with RNA integrity number (RIN) value above 6.5 was used for following library preparation. The rRNA was depleted from total RNA using Ribo-Zero rRNA Removal Kit (Bacteria) (Illumina) [51]. The ribosomal depleted RNA was then fragmented and reverse-transcribed into cDNA with random primers. The purified double-stranded cDNA by beads was then treated with End Prep Enzyme Mix to repair both ends and add a dA-tailing in one reaction, followed by a T-A ligation to add adaptors to both ends. Next generation sequencing library preparations were constructed according to the manufacturer’s protocol. The Qsep100 (Bioptic, Taiwan, China) and Qubit 3.0 Fluorometer was used to determine the quality of the libraries.

The libraries with different indices were multiplexed and sequenced on an Illumina HiSeq instrument according to manufacturer’s instructions (Illumina, San Diego, CA, USA). Sequencing was carried out by Illumina paired-end configuration. The sequencing image processing and base calling were conducted following to Illumina’s protocol on the HiSeq instrument. The read length was 90 bp, and reads were mapped to the *M. huakuii* genome using Bowtie 2. The HTSeq-count tool was used to generate the total number of uniquely mapped reads for each gene. Three independent biological replicates per sample were processed and sequenced.

### Data analysis

Differences between the average of gene expression for the control and experimental groups were analyzed by the Student’s *t*-test using SPSS software, version 18 (SPSS, Inc., Chicago, IL). For the RNA-seq study, the unique reads mapping to the *M. huakuii* genome were used for a differential gene expression analysis using the DESeq2 package. The *P*-values with false discovery rate were adjusted for multiple testing. The false discovery rate P-value < 0.001 and the absolute value of log_2_ (FC) ≥ 3 and ≤ − 3 were used to identify statistically significant changes in gene expression. For quantitative RT-PCR analysis, *p* < 0.05 was considered to be statistically significant.

## Supplementary Information

**Additional file 1: Fig. S1.** Inhibition zones of the H_2_O_2_ in disc diffusion test of different strains. *Filter paper discs* were *impregnated with* a solution containing 20 (A-D), 100 (E-H) and 250 (I-L) mg L^− 1^ of H_2_O_2_, and placed on the TY media plate on which bacteria had been spread. Plates were incubated at 28 °C for 96 h. A, E, I, *M. huakuii* 7653R; B, F, J, *M. huakuii* HKabiEi; C, G, K, *M. huakuii* HKabiEi(pBBRabiEi); D, H, L, *M. huakuii* HKabiEi(pBBR1MCS-5).

## Data Availability

Raw sequence data from these RNA-seq studies can be accessed via the NCBI Sequence Read Archive, with accession number PRJNA718999.

## Abbreviations

Abi: Bacterial abortive infection
TA: Toxin–antitoxin
T6SS: Type VI secretion system
qRT-PCR: Quantitative real-time PCR
ROS: Reactive oxygen species
NO: Nitric oxide
SEM: Scanning electron microscopy
