# Supplementary material for: The Mesorhizobium huakuii transcriptional regulator AbiEi plays a critical role in nodulation and is important for bacterial stress response
Source: BMC Microbiol. 2021 Sep 12;21:245. doi: 10.1186/s12866-021-02304-0 (PMC8436566; doi:10.1186/s12866-021-02304-0)
Supplement: Supplementary file 1 — Additional file 1: Fig. S1. Inhibition zones of the H2O2 in disc diffusion test of different strains. Filter paper discs were impregnated with a solution containing 20 (A-D), 100 (E-H) and 250 (I-L) mg L− 1 of H2O2, and placed on the TY media plate on which bacteria had been spread. Plates were incubated at 28 °C for 96 h. A, E, I, M. huakuii 7653R; B, F, J, M. huakuii HKabiEi; C, G, K, M. huakuii HKabiEi(pBBRabiEi); D, H, L, M. huakuii HKabiEi(pBBR1MCS-5). [file 12866_2021_2304_MOESM1_ESM.docx]

Additional file 1


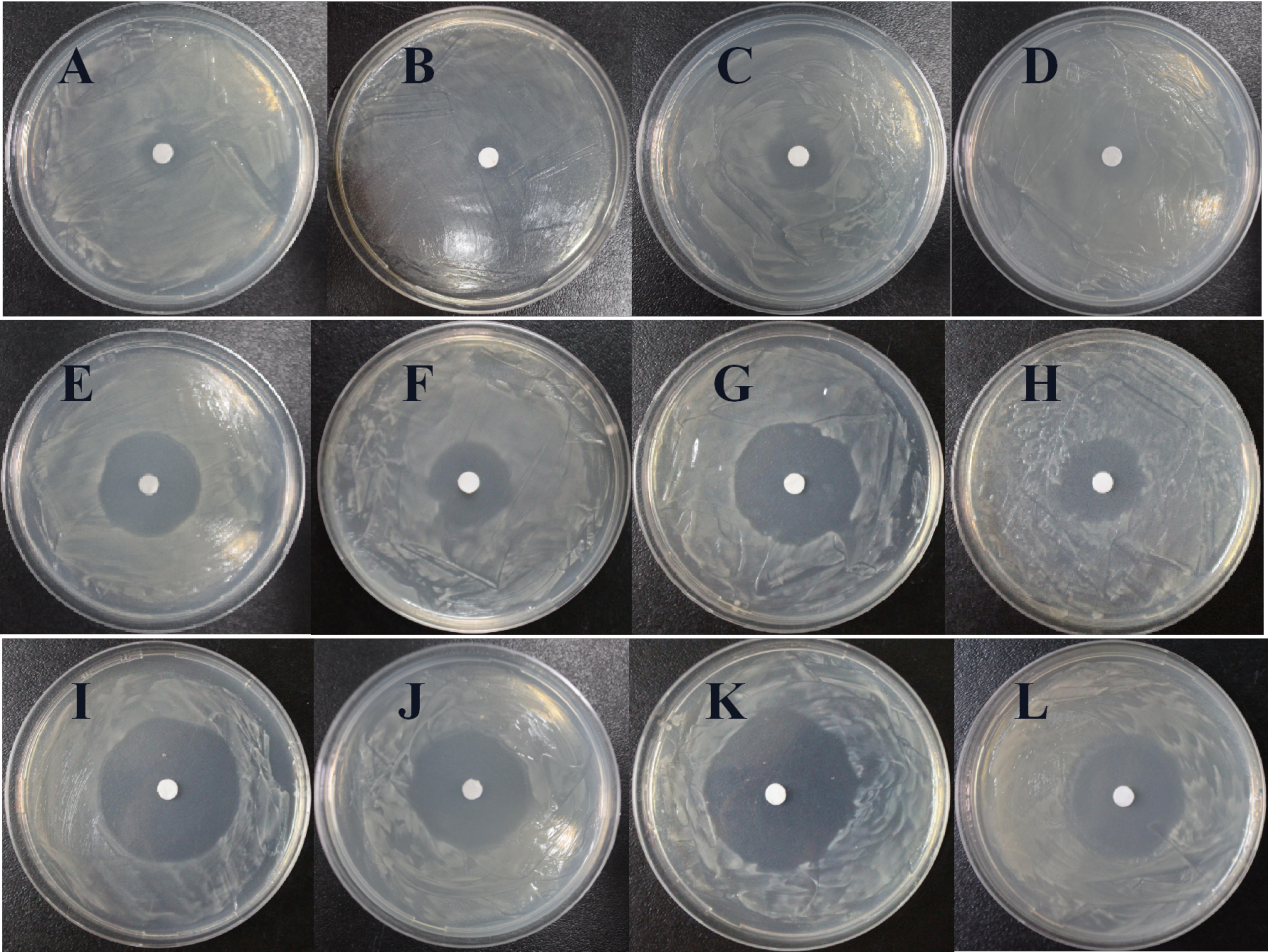


Fig. S1. Inhibition zones of the H_2_O_2_ in disc diffusion test of different strains. Filter paper discs were impregnated with a solution containing 20 (A-D), 100 (E-H) and 250 (I-L) mg L^-1^ of H_2_O_2_, and placed on the TY media plate on which bacteria had been spread. Plates were incubated at 28°C for 96 h. A, E, I, *M. huakuii* 7653R; B, F, J, *M. huakuii* HKabiEi; C, G, K, *M. huakuii* HKabiEi(pBBRabiEi); D, H, L, *M. huakuii* HKabiEi HKabiEi(pBBR1MCS-5).
